# Recent Advances and Future Perspective in Microbiota and Probiotics

**DOI:** 10.1155/2015/275631

**Published:** 2015-04-23

**Authors:** Haruki Kitazawa, Susana Alvarez, Alexander Suvorov, Vyacheslav Melnikov, Julio Villena, Borja Sánchez

**Affiliations:** ^1^Food and Feed Immunology Group, Laboratory of Animal Products Chemistry, Graduate School of Agricultural Science, Tohoku University, Sendai 981-8555, Japan; ^2^Laboratory of Immunobiotechnology, CERELA-CONOCET, 4000 Tucuman, Argentina; ^3^Department of Molecular Microbiology, Institute of Experimental Medicine, Saint Petersburg 197376, Russia; ^4^International Science and Technology Center (ISTC), Moscow 127374, Russia; ^5^Department of Microbiology and Biochemistry of Dairy Products, IPLA-CSIC, 32004 Ourense, Spain

Recent studies have highlighted the critical role of intestinal microbes on health. The various bacterial communities in the gut have many functions including metabolic, barrier effect, and trophic and immunological functions. The gut microbiota therefore performs a large number of important roles that define the physiology of the host. Advances in the understanding of microbiota interaction with the host have irrevocably altered the view of mammalian metabolism and gut biology. As stated by Kinross et al. [1], “*human gut biology and metabolism is not only influenced by the human genome, but a core gut microbiome exist within the human gut, at least at a genomic or metabolic level, and this is fundamental to the maintenance of health, the development of disease and human metabolic processes.”*

The understanding of the gut microbiota and its activities is essential for the generation of future personalized healthcare strategies. In this regard, there is a growing body of evidence to support the potential use of selected bacterial strains in the prevention and treatment of various human and animal diseases. Numerous studies including different probiotic strains have been performed in humans and animal models to investigate their beneficial effects [2–4]. Overall there is encouraging evidence that specific probiotic strains are valuable in the prevention and treatment of different diseases and their successful application is related to the better understanding of the cellular and molecular mechanisms of probiotic action.

Studies have provided insight into the mechanisms by which probiotic bacteria are able to regulate the colonization and eradication of pathogens in the gut, including competition for limited nutrients in the intestine and modulation of the mucosal immune system [4, 5]. In addition, it has been well established that probiotics are an important prophylactic or therapeutic strategy for many mucosal and nonmucosal immune-related conditions, such as inflammatory bowel diseases (IBDs), celiac disease, metabolic syndrome, and diabetes [6]. In this regard, results from experiments in animal models of IBDs overwhelmingly support a causal role of the microbiota in these diseases. In this special issue, A. Hevia et al. explored the levels of antibodies raised against extracellular proteins produced by different food bacteria from the genera* Bifidobacterium* and* Lactobacillus*, in healthy individuals and IBDs patients. The authors found that IBD patients appeared to have different immune responses to food bacteria. The work could set the basis for developing systems for early detection of IBD, based on the association of high levels of antibodies developed against extracellular proteins from lactic acid bacteria. On the other hand, data from animal models of colitis have indicated that specific probiotic* Lactobacillus* and* Bifidobacterium* strains could prevent and treat intestinal inflammation. The study of R. Chauhan et al. was undertaken to evaluate the antioxidative potentials of* Lactobacillus fermentum* Lf1, a promising indigenous probiotic* Lactobacillus* strain, to manage oxidative stress and modulation of lipid peroxidation* in vitro* and* in vivo. *The authors showed in intestinal epithelial cells cultures and in a DSS colitis mouse model that the probiotic strain Lf1 was able to increase the expression of antioxidative enzymes and reduce colitis, indicating that probiotics could be explored as a new strategy for IBD management through activation of the antioxidant enzyme system. In addition, J. Breton et al. studied the direct immune responses to alimentary fibers in murine model of experimental colitis. The study strongly suggests that intrinsic, nonprebiotic-driven effects of selected oligosaccharide and polysaccharide fibers can influence immunomodulatory functions and that these fibers could be used to enhance dietary interventions for the treatment of inflammatory disorders such as IBD and other diseases with an immune component. The use of fibers alone or in combination with selected probiotics (symbiotic preparations) could be considered as a promise intervention tool for inflammatory diseases.

Fermented dairy foods result from the metabolic activity of complex and heterogeneous bacterial communities; then these dairy fermented products contain a complex, live microbial consortium mostly represented by lactic acid bacteria, which enter the human body and reach the gastrointestinal tract, where they can transiently interact with the resident gut microflora of the host. The interplay between these two microbial communities can greatly contribute to human health. However, evaluation of the interaction between these microbial populations has the obstacle of the lack of simple model organisms suitable for these studies. In this special issue, the work of E. Zanni et al. tested the nematode* Caenorhabditis elegans *as a simple animal model to evaluate the effects of a complex food-derived microbiota on well characterized metabolic pathways. Authors provide evidence that feeding* C. elegans* with a lactic acid bacteria consortium influences longevity, larval development, fertility, lipid accumulation, and gene expression related to obesity in this model organism, as supported by transcriptional analysis of some genes involved in fat metabolism. The work clearly demonstrates the applicability of* C. elegans* model in the field of host-microbiota interaction.

Currently, the use of genetically modified commensal and lactic acid bacteria to deliver compounds of health interest is gaining importance as an extension of the probiotic concept [7]. Most of the works using recombinant friendly bacteria are mainly related to vaccines. Several antigens from pathogens have been expressed in genetically engineered lactic acid bacteria and these recombinant bacteria have been successfully used for inducing protective immunity in animal models. However, recombinant friendly bacteria can be also used for exploring novel effective strategies to deliver therapeutic molecules to the mucosal tissues in order to avoid degradation. In this special issue P. Kumar et al. studied the potential beneficial effects of* E. coli *16 expressing* Vitreoscilla *hemoglobin gene, associated with bacterial respiration under microaerobic condition, on carbon tetrachloride induced toxicity in rats. The work showed that the presence of* Vitreoscilla *hemoglobin gene improved the growth and intestinal tract colonization of* E. coli* 16. Moreover, recombinant* E. coli *16 enhanced catalase activity in rats, prevented absorption of carbon tetrachloride in the intestine, and ameliorated hepatotoxicity. On the other hand, S. Shigemori et al. developed a *β*-lactoglobulin-secreting* Lactococcus lactis* and demonstrated that this recombinant strain is able to inhibit dipeptidyl peptidase-IV (DPP-IV) activity. DPP-IV is a serine protease and its endogenous physiological substrates are incretins. The incretins are primarily glucose-dependent insulinotropic polypeptide and glucagon-like peptide-1, which drive insulin secretion in pancreatic *β* cells and suppress pancreatic glucagon production. Thus, DPP-IV inhibitors are used in the management of type 2 diabetes mellitus.

In summary, this special issue covers a range of diverse topics related to microbiota and probiotic in gut health and disease, thus highlighting the potential beneficial role friendly bacteria in human health. We hope the papers published will serve to further highlight the potential application of probiotics for the prevention and treatment of gut diseases, as well as stimulating further research into the cellular and molecular mechanisms of probiotic actions.

*Haruki Kitazawa* *Susana Alvarez* *Alexander Suvorov* *Vyacheslav Melnikov* *Julio Villena* *Borja Sánchez*
